# Pisa Syndrome in Chinese Patients With Parkinson's Disease

**DOI:** 10.3389/fneur.2019.00651

**Published:** 2019-06-20

**Authors:** Kuncheng Liu, Ruwei Ou, Qianqian Wei, Bei Cao, Yongping Chen, Wei Song, Ying Wu, Huifang Shang

**Affiliations:** Department of Neurology, West China Hospital, Sichuan University, Chengdu, China

**Keywords:** parkinson's disease, pisa syndrome, prevalence, age, levodopa equivalent daily doses, motor symptoms

## Abstract

**Objective:** To investigate the prevalence and the clinical factors related to Pisa syndrome (PS) in Chinese Parkinson's disease (PD) patients.

**Methods:** A total of 2,167 PD patients were continuously included in this observational study. Patients with PS were identified as presented with a lateral trunk flexion of at least 10° that can be completely alleviated by passive mobilization or supine positioning. The data of the motor and non-motor symptoms including depression, anxiety and cognitive dysfunction was collected and analyzed.

**Results:** We found seventy-seven (3.6%) PD patients presenting with PS. The following variables including age, disease duration, levodopa equivalent daily doses (LEDD), the proportion of males, the proportion of participants using levodopa, dopaminergic agonist, amantadine and entacapone, the proportion of motor fluctuations, scores of Hamilton Depression Scale (HAMD), Hamilton Anxiety Scale (HAMA), Unified PD Rating Scale (UPDRS) part III, and Hoehn and Yahr (H&Y) stage were significantly higher in patients with PS compared with patients without PS (*P* < 0.05). Scores of the Frontal Assessment Battery (FAB) and the Montreal Cognitive Assessment (MoCA) were not different between the two groups. The binary logistic regression model indicated that the presence of PS was associated with older age (OR = 1.027, *P* = 0.030), higher LEDD (OR = 1.002, *P* < 0.001) and a higher UPDRS III score (OR = 1.060, *P* < 0.001), but had no relationship with HAMD and HAMA scores.

**Conclusion:** PS is relatively rare (3.6%) in Chinese PD patients. It is likely associated with older age, higher LEDD and more severe motor disabilities. However, non-motor symptoms such as depression, anxiety, and cognitive dysfunction have no association with PS in PD. These findings provided important complementary information for identifying the underlying mechanisms of PS.

## Introduction

Pisa Syndrome (PS) was first systematically described in 1972 among patients taking antipsychotics (1). It is a postural deformity defined as a marked lateral trunk flexion which can be aggravated by standing, sitting, or walking and completely alleviated by passive mobilization or supine position (2). There is no consensus on the diagnostic criteria for PS. The most commonly used diagnostic criterion for PS is based on a lateral trunk flexion of at least 10° (2), and PS can be divided into mild (<20°) or severe (≥20°) according to the angle of lateral trunk flexion (3).

PS can be seen in a variety of neurological diseases, and it was first reported in 2003 among patients with Parkinson's disease (PD) (4). PS is a disabling and potential reversible symptom which severely impacts on the quality of life in PD patients. However, the pathogenesis of PS remains unclear, although several hypotheses point out that it may be a result of a multifactorial process involving both central and peripheral mechanisms (5, 6).

The clinical characteristics of PS in patients with PD were poorly covered. It is reported that the prevalence of PS in Caucasian patients with PD ranged from 1.9% (7) to 16.5% (3). Only one Chinese study with 503 PD patients was conducted to explore the prevalence and characteristics of the coronal plane deformity (8). However, the inclusion criteria of the study (8) did not point out whether the patients' lateral trunk flexion were fixed or mobile since the most important differential diagnosis for PS is scoliosis, which does not resolve in the lying position. Although the authors found that PD patients with PS were older and in more severe stage and longer disease duration in this study (8), their findings were not further analyzed by multifactor regression models. Thus, whether there was a robust association between these factors and PS or not was still unclear. Besides, the findings of the study (8) were not supported by a Caucasian research (9).

In addition, the association between PS and non-motor symptoms in PD remains unknown. Only one study (10) found that PD patients with PS performed worse on attention and visuoperceptual tests but had no difference in depression tests when compared with patients without PS. The association between PS and anxiety in PD has not been explored yet. Therefore, we planned to perform a large sample study to investigate the prevalence and the clinical factors related to PS in Chinese PD patients.

## Patients and Methods

A total of 2,167 PD patients (1,174 males and 993 females) were continuously included in the observational study between August 2012 to March 2018 from the Department of Neurology, West China Hospital, Sichuan University. All participants were from the neurological in-patient ward or the outpatient clinic. All subjects gave written informed consent in accordance with the Declaration of Helsinki. The study was approved by the Ethics Committee of West China Hospital of Sichuan University. All PD patients included in this study were diagnosed according to the United Kingdom PD Society Brain Bank Clinical Diagnostic Criteria for PD. Exclusion criteria were listed as follows: (i) concomitant neurological or non-neurological diseases known to determine postural deformities such as primary dystonia, myopathy, myositis, idiopathic scoliosis, ankylosing spondylitis, or rheumatoid arthritis; (ii) a history of major spinal/vertebral surgery; (iii) treatment with drugs potentially able to induce PS such as antipsychotics or antiemetics in the last 6 months prior to enrolment; (iv) presented with secondary parkinsonism or atypical parkinsonism including multiple system atrophy (MSA), progressive supranuclear palsy (PSP), corticobasal degeneration (CBD) (The diagnoses of MSA, PSP, and CBD were based on the published diagnostic criteria for MSA (11), PSP (12), and CBD (13), respectively.); (v) years of education ≤ 3; (vi) presence of dementia associated with PD as defined by clinical criteria (14) or cognitive status was considered insufficient to cooperate with investigators; (vii) condition was too severe to complete the assessment protocol such as unable to stand unsupported.

**Figure 1:**
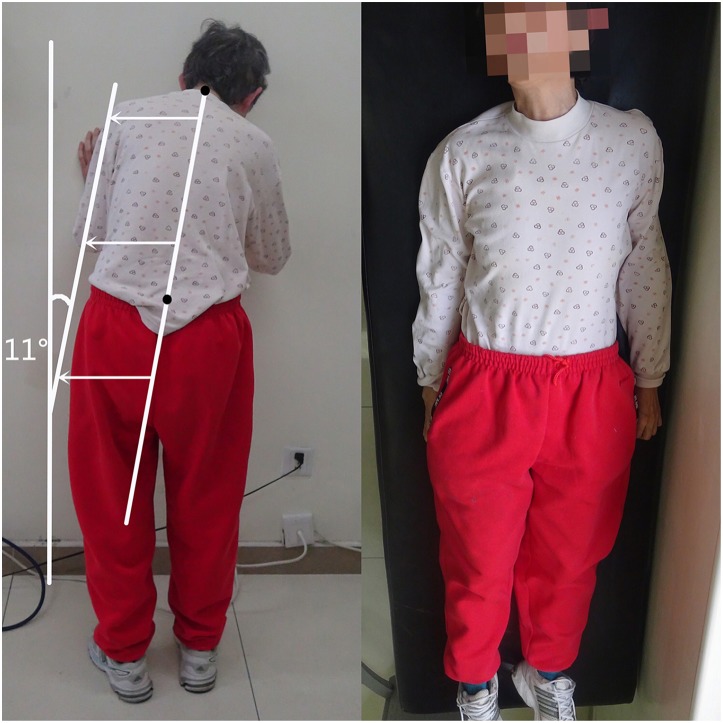
A PD patient with PS exhibited a lateral trunk flexion of 11° when standing and completely alleviated in the supine position. The written informed consent has been obtained from the individual for the publication of this image.

All recruited PD patients underwent a measurement for the angle of the lateral flexion of the trunk with a wall goniometer and were classified into two groups, the presence or absence of PS, according to the commonly used criteria for PS (2). The medication states of patients when they underwent the measurements are listed in Table 1. If a patient presented with a lateral trunk flexion of at least 10°, and this postural abnormality can be entirely alleviated by passive mobilization or supine positioning, he/she was identified as the presence of PS (2) (Figure 1).

**Table 1:** Demographic and clinical features between PD patients with and without PS.

	**Total**	**With PS**	**Without PS**	**Test**	***P*-value**
Number of patients	2,167	77	2,090		
Sex (male)	1,174 (54.2%)	52 (67.5%)	1,122 (53.7%)	2	0.017^*^
Mean age (years)	61.0 ± 11.5	66.7 ± 9.0	60.8 ± 11.5	1	<0.001^*^
Mean age of onset (years)	56.9 ± 11.7	59.3 ± 10.6	56.8 ± 11.7	1	0.059
EOPD	345 (15.9%)	8 (10.4%)	337 (16.1%)	2	0.177
Education level (years)	9.8 ± 4.2	9.9 ± 4.4	9.8 ± 4.1	1	0.779
Disease duration (years)	4.1 ± 4.3	7.3 ± 5.7	4.0 ± 4.2	1	<0.001^*^
Family history	251 (11.6%)	6 (7.8%)	245 (11.7%)	2	0.290
Hyposmia	790 (36.5%)	34 (44.2%)	756 (36.2%)	2	0.153
LEDD (mg/day)	299.9 ± 294.6	544.3 ± 360.6	290.9 ± 288.0	1	<0.001^*^
Use of levodopa	1,319 (60.9%)	65 (84.4%)	1,254 (60.0%)	2	<0.001^*^
Use of dopaminergic agonist	767 (35.4%)	41 (53.2%)	726 (34.7%)	2	0.001^*^
Use of amantadine	420 (19.4%)	30 (39.0%)	390 (18.7%)	2	<0.001^*^
Use of anticholinergic agents	180 (8.3%)	8 (10.4%)	172 (8.2%)	2	0.500
Use of entacapone	101 (4.7%)	10 (13.0%)	91 (4.4%)	5	0.002^*^
Use of selegiline	33 (1.5%)	1 (1.3%)	32 (1.5%)	5	1.000
UPDRS part III	28.7 ± 13.9	44.2 ± 14.2	28.1 ± 13.6	3	<0.001^*^
Medication state (“ON”)	795 (36.7%)	32 (41.6%)	763 (36.5%)	2	0.366
H&Y stage	2.2 (0.5)	2.8 (1.0)	2.2 (0.5)	4	<0.001^*^
1.0	236 (10.9%)	0	236 (11.3%)		
1.5	133 (6.1%)	0	133 (6.3%)		
2.0	1,038 (47.9%)	23 (29.9%)	1,015 (48.6%)		
2.5	375 (17.3%)	21 (27.2%)	354 (16.9%)		
3.0	289 (13.3%)	20 (26.0%)	269 (12.9%)		
4.0	84 (3.9%)	11 (14.3%)	73 (3.5%)		
5.0	12 (0.6%)	2 (2.6%)	10 (0.5%)		
**Motor Complication**					
Fluctuation	278 (12.8%)	29 (37.7%)	249 (11.9%)	2	<0.001^*^
Dyskinesia	131 (6.0%)	8 (10.4%)	123 (5.9%)	2	0.136
FAB	15.4 ± 2.5	14.6 ± 2.7	15.4 ± 2.5	3	0.262
MoCA	23.4 ± 4.7	22.1 ± 4.9	23.5 ± 4.7	3	0.235
HAMD	10.5 ± 8.8	14.1 ± 8.9	10.3 ± 8.8	3	0.002^*^
HAMA	7.7 ± 6.8	10.3 ± 7.6	7.6 ± 6.8	3	0.005^*^

*PD, Parkinson's disease; PS, Pisa syndrome; EOPD, early-onset Parkinson's disease, LOPD, late-onset Parkinson's disease; LEDD, levodopa equivalent daily doses; UPDRS, Unified PD Rating Scale; H&Y stage, Hoehn and Yahr stage; FAB, Frontal Assessment Battery; MoCA, Montreal Cognitive Assessment; PDQ-39, the 39-item Parkinson's Disease Questionnaire; HAMD, Hamilton Depression Scale; HAMA, Hamilton Anxiety Scale*.

*Test 1: Student T-test*.

*Test 2: Chi-square test*.

*Test 3: ANCOVA test with adjustment for sex, age, age of onset and disease duration*.

*Test 4: Mann-Whitney U-test*.

*Test 5: Fisher exact test*.

* *Significant difference*.

Demographic and clinical data including age, sex, age of onset, disease duration, education level, family history of PD, hyposmia, treatment regimen, levodopa equivalent daily doses (LEDD) and motor complications were collected by professional neurologists specializing in movement disorders, through a face-to-face interview. The LEDD was calculated by the commonly used protocol given by Tomlinson et al. (15). Early-onset PD (EOPD) was defined as onset age ≤45 years old and late-onset PD (LOPD) as onset age > 45 years old.

Also, all participants underwent a series of detailed clinical assessments. The Unified PD Rating Scale (UPDRS) part III (16) and the Hoehn and Yahr (H&Y) stage (17) were used to evaluate the motor disability. For analytical purposes, part of the UPDRS part III items that reflects asymmetry of parkinsonian signs was derived as the asymmetry subscore, including tremor at rest in upper and lower extremities, action or postural tremor in hands, rigidity in upper and lower extremities, finger taps, hand movements, rapid alternating movements of hands and leg agility, and was calculated as the total value of the differences between sides of each item (9). To further investigate the severity of cognition, depression, anxiety symptoms on the development of PS, we applied additional specific scales to evaluate these conditions. Cognitive function was assessed using the Frontal Assessment Battery (FAB) (18) and the Montreal Cognitive Assessment (MoCA) (19). The severity of depression was assessed using the Hamilton Depression Scale (HAMD; 24 items) (20), while the severity of anxiety was evaluated using the Hamilton Anxiety Scale (HAMA) (21). Meanwhile, the 39-item PD Questionnaire (PDQ-39) (22) was used to evaluate the quality of life of PD patients.

## Statistical Analyses

All analyses were performed using SPSS 21.0. All statistical tests were two-tailed and *P*-values < 0.05 were considered statistically significant. Continuous data were presented as mean ± standard deviation and were analyzed by Student's *T*-test or analyses of covariance (ANCOVAs) with adjustment for confounding factors. Categorical data were presented as counts (percentages) and were analyzed by Chi-square test or Fisher's exact test. Ranked data (H&Y stage) were presented as the median values (quartile) and was analyzed by Mann-Whitney *U*-test. Finally, a forward stepwise binary logistic regression model was performed to explore the factors associated with the development of PS. Presence or absence of PS was employed as the dependent variable. Age, sex, disease duration, use of levodopa, use of dopaminergic agonist, use of amantadine, use of entacapone, LEDD, UPDRS part III score, motor fluctuation, the HAMD score, and the HAMA score were employed as co-variables, which were selected according to the significant results (selection criteria *P* < 0.05) from comparisons between PD patients with and without PS.

## Results

The demographic and clinical features of included PD patients are listed in Table 1. Among the 2,167 PD patients, 77 (52 males and 25 females, 3.6%) patients presented with PS at the time of evaluation. PD patients with PS were older, had a significantly higher proportion of male sex, a longer disease duration, higher LEDD, a greater H&Y stage and more frequent motor fluctuations when compared with PD patients without PS (*P* < 0.05). The proportion of participants using levodopa, dopaminergic agonist, amantadine, and entacapone in PD patients with PS was significantly higher than those without PS (*P* < 0.05). After adjusted for sex, age, age of onset and disease duration, compared with PD patients without PS, PD patients with PS presented with higher scores of HAMD, HAMA, and UPDRS part III (*P* < 0.05). No significant differences in the mean age of onset, the ratio of EOPD and LOPD, education level, family history, hyposmia, dyskinesia, and use of anticholinergic agents or selegiline were found between the two groups. Comparisons of the UPDRS part III asymmetry subscore between PD patients with and without PS are presented in Table 2. There were no differences in neither the total asymmetry score nor subscores of each item between the two groups.

**Table 2:** Asymmetry subscore derived from the UPDRS part III items between PD patients with and without PS.

	**Total**	**With PS**	**Without PS**	***P*-value**
Total asymmetry	2.4 ± 2.6	2.2 ± 2.4	2.4 ± 2.5	0.740
Tremor at rest in upper and lower extremities	1.0 ± 1.2	0.8 ± 1.0	1.0 ± 1.2	0.671
Action or postural tremor in hands	0.4 ± 0.6	0.3 ± 0.7	0.4 ± 0.6	0.242
Rigidity in upper and lower extremities	1.0 ± 1.1	0.7 ± 0.8	1.0 ± 1.1	0.688
Finger taps	0.5 ± 0.6	0.4 ± 0.6	0.5 ± 0.6	0.881
Hand movements	0.5 ± 0.6	0.4 ± 0.5	0.5 ± 0.6	0.833
Rapid alternating movements of hands	0.5 ± 0.6	0.4 ± 0.6	0.5 ± 0.6	0.946
Leg agility	0.4 ± 0.5	0.3 ± 0.4	0.4 ± 0.5	0.438

*UPDRS, Unified Parkinson's Disease Rating Scale; PD, Parkinson's disease; PS, Pisa syndrome*.

*P-value was calculated from an ANCOVA test with adjustment for sex, age, age of onset, and disease duration*.

Comparisons of cognitive assessment results between PD patients with and without PS are presented in Table 3. The FAB score, MoCA score and its subscores were not significantly different between the two groups.

**Table 3:** Cognitive assessments between PD patients with and without PS.

	**Total**	**With PS**	**Without PS**	***P*-value**
FAB	15.4 ± 2.5	14.6 ± 2.7	15.4 ± 2.5	0.262
MoCA	23.4 ± 4.7	22.1 ± 4.9	23.5 ± 4.7	0.235
Visuospatial/executive abilities	3.5 ± 1.5	3.2 ± 1.4	3.5 ± 1.5	0.535
Naming	2.5 ± 0.8	2.5 ± 0.9	2.5 ± 0.8	0.532
Attention	5.3 ± 1.0	5.1 ± 1.1	5.3 ± 1.0	0.185
Language	2.0 ± 1.0	1.9 ± 1.0	2.0 ± 1.0	0.884
Abstraction	1.1 ± 0.7	1.0 ± 0.7	1.1 ± 0.7	0.053
Memory	2.7 ± 1.7	2.2 ± 1.6	2.7 ± 1.7	0.226
Orientation	5.7 ± 0.7	5.6 ± 0.7	5.7 ± 0.7	0.647

*PD, Parkinson's disease; PS, Pisa syndrome; FAB, Frontal Assessment Battery; MoCA, Montreal Cognitive Assessment*.

*P-value was calculated from an ANCOVA test with adjustment for sex, age, age of onset and disease duration*.

Comparisons of the quality of life between PD patients with and without PS are presented in Table 4. PD patients with PS had a significantly higher score in the “emotional well-being” subdomain of the PDQ-39 than those without PS (*P* < 0.05). However, the PDQ-39 score was not significantly different between the two groups.

**Table 4:** Quality of life between PD patients with and without PS.

	**Total**	**With PS**	**Without PS**	***P*-value**
PDQ-39	32.5 ± 24.2	45.1 ± 27.6	32.0 ± 24.0	0.195
Mobility	9.0 ± 9.7	15.1 ± 11.9	8.8 ± 9.6	0.866
Activities of daily living	5.4 ± 5.4	9.0 ± 6.7	5.3 ± 5.3	0.562
Emotional well-being	5.5 ± 5.3	5.7 ± 5.2	5.5 ± 5.3	0.030^*^
Stigma	3.2 ± 4.1	3.5 ± 4.4	3.2 ± 4.1	0.279
Social support	0.9 ± 1.8	0.7 ± 1.5	0.9 ± 1.8	0.141
Cognition	4.0 ± 3.0	5.0 ± 3.4	4.0 ± 3.0	0.453
Communication	1.5 ± 2.1	2.3 ± 2.6	1.5 ± 2.1	0.187
Bodily discomfort	3.0 ± 2.6	3.7 ± 2.7	3.0 ± 2.6	0.841

PD, Parkinson's disease; PS, Pisa syndrome; PDQ-39, the 39-item Parkinson's Disease Questionnaire

*P-value was calculated from an ANCOVA test with adjustment for sex, age, disease duration and UPDRS part III score*.

* *Significant difference*.

The potential risk factors of PD patients developing PS are presented in Table 5. The binary logistic regression model indicated that older age (OR = 1.027, *P* = 0.030), higher LEDD (OR = 1.002, *P* < 0.001) and a higher UPDRS III score (OR = 1.060, *P* < 0.001) were associated with the presence of PS.

**Table 5:** Regression analyses of clinical variables associated with PS in PD.

**Independent significant covariates**	**OR (95%CI)**	***P*-value**
Mean age	1.027 (1.003–1.053)	0.030
LEDD	1.002 (1.001–1.002)	<0.001
UPDRS part III	1.060 (1.043–1.077)	<0.001

*PD, Parkinson's disease; PS, Pisa syndrome; LEDD, levodopa equivalent daily doses; UPDRS, Unified PD Rating Scale*.

*P-value was calculated from a binary regression analyses model, with age, age of onset, sex, disease duration, use of levodopa, use of dopaminergic agonist, use of amantadine, use of entacapone, LEDD, UPDRS part III score, motor fluctuation, the HAMD score, the HAMA score and the MoCA “abstraction” subscore were included as co-variables, and presence/absence of PS was set as dependent variable*.

## Discussion

To the best of our knowledge, this is the largest sample size study to investigate the prevalence and clinical characteristics of PS in Chinese PD population. The prevalence of PS in Chinese PD patients (3.6%) is relatively low. We found PS was associated with older age, higher LEDD and more severe motor disabilities. However, non-motor symptoms such as depression, anxiety, and cognitive dysfunction have no association with PS in PD.

The low prevalence of PS in our PD patients (3.6%) is similar to the findings of an Indian study and an Italian study (1.9% and 4.3%, respectively) (7, 23), but lower than the result of another Italian research (8.8%) (3). Such difference may be due to the differences in diagnostic criteria for PS [the angle of the lateral trunk flexion varies from 5° (7) to 10° (3, 23)], sample size and genetic background among studies.

In our study, PS in PD patients is associated with older age, which was in agreement with two previous studies (3, 8). Meanwhile, the mean age survived the multivariate logistic regression analysis. There have been some studies reporting that many PD patients with postural deformities had a history of degenerative spinal conditions and other medical conditions associated with gerontism such as osteoporosis and arthrosis (2, 6), and these associations were also confirmed in PS (3). We can assume that older PD patients have a higher risk of developing osteoarthropathies like osteoporosis and arthrosis, and these comorbidities make older PD patients more likely to suffer from PS. Medical conditions involving the musculoskeletal system may play a role in the peripheral mechanism of the pathophysiology of PS, and this deserves further investigation.

In the present study, we found that PD patients with PS had a substantially longer disease duration and presented with more severe motor disabilities and more frequent motor fluctuation, which suggested that PS in PD patients may occur in more advanced disease stages. Our findings are supported by several previous studies (3, 5, 8), although one study failed to find such differences (9). In addition, the multivariate logistic regression found that motor disability was an independent risk factor associated with PS. Rigidity, one of the major manifestations in PD, has been referred to a common mechanism causing postural abnormalities in PD patients (24). In the advanced PD stages, the striatum is severely denervated and patients are likely to suffer from more serious motor disability, including rigidity, and abnormal postures such as PS may emerge (25). Additionally, we found that PS patients were receiving higher LEDD and taking more levodopa, dopaminergic agonist, amantadine, and entacapone. LEDD has been identified as an independent risk factor for PS by the multivariate logistic regression analysis. Higher dosage of dopaminergic drugs also indicates that PS may occur in more advanced stages. Dopaminergic drug therapy might promote the development of PS by priming the basal ganglia circuit (26). Priming is a pharmacological phenomenon which is the hypersensitivity of dopaminergic receptors after denervation of the nigrostriatal pathway such as in PD (26). Higher dopamine exposure might lead to a more sensitive response in the more affected striatum, promoting PS onset in a high-risk population (26).

Previous studies (9, 27) have proposed a hypothesis that the imbalance of basal ganglia loop is a primary central mechanism in the pathogenesis of PS since PD patients with PS showed greater motor asymmetry than PD patients without PS. However, no differences in the asymmetry of parkinsonian signs were found between the two groups in our study, which was in accordance with some previous studies (2, 25). The only pathological study (28) of PS in PD patients found no asymmetry in the loss of dopaminergic neurons in the basal ganglia. These findings indicate a more widespread alteration of central structures and neurotransmitters such as serotonin (5-HT) and noradrenaline (29) other than the asymmetry of basal ganglia outflow in PS development.

As for non-motor symptoms in PD, a European study found a correlation between PS and attention/visuoperceptual deficits in patients with PD (10), while there was no significant difference in cognitive function between PD patients with and without PS in our study. This discrepancy may be due to the fact that the European study utilized more specific neuropsychological batteries including Rey's words list (RAVLT), Stroop test, Trail Making Test (TMT), constructional apraxia test (CA) and Benton's judgment of line orientation test (BJLOT) to test cognitive function. Therefore, further studies utilizing more detailed scales of cognition will be helpful to clarify this question.

Moreover, our PD patients with PS presented more severe depression and anxiety symptom according to the HAMD and HAMA results, while a previous study did not find such a significant difference (10), which may attribute to the relatively small size of that study population (20 patients with PS and 20 patients without PS). However, the HAMD and HAMA score did not survive the multivariate logistic regression analysis, which means depression and anxiety were not potential risk factors for PS in PD. Although there was no association between depression, anxiety and PS in PD, the fact that PD patients with PS presented more severe depression and anxiety symptom suggests non-motor symptoms should be treated when managing PD patients with PS.

A previous study showed that PD patients with PS had a reduced quality of life by the scale of PDQ-39 (8). However, our research found no significant difference in the total score of PDQ-39 between PD patients with and without PS, which may be due to some PD patients with PS were too severe to be able to complete the assessment scales and to recruit into the study. Nevertheless, PD patients with PS had a significantly higher score in the “emotional well-being” subdomain of the PDQ-39 than those without PS, which is concordant with the HAMD and HAMA results in this study that PD patients with PS suffered more from negative emotions like depression and anxiety.

Previous studies have found that muscle atrophy and fatty degeneration, investigated by magnetic resonance imaging (MRI) and computerized tomography (CT), can occur in both sides of the trunk in PD patients with PS, which might be caused by peripheral changes such as stretching stress on the muscle, decreased range of movement and increased load on the spine and/or lower limbs (9, 30), suggesting a peripheral mechanism involvement. Together with our study, it seems that both central and peripheral mechanisms may be involved in the development of PS. Overlap of central and peripheral pathways may explain the clinical heterogeneity and prognostic differences of PS. Further studies including investigations into both central and peripheral mechanisms (e.g., electromyography) are needed to elucidate the pathophysiology of PS.

The current study has several limitations. First, we did not recruit patients who were too severely affected to complete the assessment protocol, unable to stand unsupported for example, which may be the reason why there was no difference in the total score of PDQ-39 between patients with and without PS. Second, other potential factors such as gait, which has contradictory findings among studies (27, 31), were not specifically analyzed in this study. Finally, we did not identify the risk factors of PS in different medication states (“OFF” or “ON”), so the role of drug therapy in the pathophysiology of PS was difficult to explain.

## Conclusion

PS is relatively rare (3.6%) in Chinese PD patients. It is likely associated with older age, higher LEDD and more severe motor disabilities. However, non-motor symptoms such as depression, anxiety, and cognitive dysfunction have no association with PS in PD. This provided important complementary information for identifying the underlying mechanisms of PS.

## Data Availability

All datasets generated for this study are included in the manuscript and/or the supplementary files.

## Ethics Statement

All subjects gave written informed consent in accordance with the Declaration of Helsinki. The study was approved by the Ethics Committee of West China Hospital of Sichuan University.

## Author Contributions

KL and RO designed the study and drafted the manuscript. QW, YC, WS, and YW did the investigation, performed measurements, and collected the data. BC did the statistical analysis. HS reviewed and edited the manuscript.

### Conflict of Interest Statement

The authors declare that the research was conducted in the absence of any commercial or financial relationships that could be construed as a potential conflict of interest.
